# Idiopathic intussusception in an adult—an imageguided diagnosis

**DOI:** 10.1259/bjrcr.20150508

**Published:** 2016-05-05

**Authors:** Vivienne Nkechi Eze

**Affiliations:** General Surgery, Russells Hall Hospital, Dudley, UK

## Abstract

A 22-year-old female with no significant past medical history presented with a short history of sudden onset, central abdominal pain. She was investigated with ultrasound scan of her abdomen, which showed a “lumen in lumen” appearance of the small bowel suggestive of intussusception. Subsequent CT scan of the abdomen confirmed the diagnosis with characteristic appearances of “target” sign and “reniform” mass confirming intussusception and suggestive of consequent bowel ischaemia. She went on to have an emergency laparotomy and right hemicolectomy. No lead points were identified intraoperatively and histopathology results showed no benign or malignant tumours and no polyps in the bowel lumen. She recovered well postoperatively and was discharged. Diagnosing intussusception in adults can be challenging and this case discusses the different imaging modalities that can be used in diagnosis with images of associated pathognomonic signs.

## Background

Intussusception is a rare cause of abdominal pain in adults and is seldom suspected in adults presenting with acute onset abdominal pain. Pre-operative diagnosis is often challenging, leading to delayed diagnosis and inevitable bowel ischaemia. This case highlights the role and importance of imaging in the diagnosis and management of such individuals.

## Case presentation

A 22-year-old female presented with a 7-h history of sudden onset central abdominal pain. The pain was constant and unremitting, with associated nausea, followed by a few episodes of vomiting. She also reported reduced appetite. She denied any change in bowel habits and any urinary tract symptoms and had passed bowel motions with no difficulty earlier that day. She was previously well, with no previous medical problems or allergies. She was not a smoker and only drank alcohol occasionally. On admission, her pulse was 63 beats min^–1^ with a regular rhythm, temperature was 35.9 ^o^C, blood pressure was 132/73 mmHg and her respiratory rate was 24 breaths min^–1^. On examination, she had a soft, non-distended abdomen with tenderness around her umbilical and suprapubic region. She also had fullness in her suprapubic region, thought to be a distended bladder. Initial blood tests showed normal liver and renal function (estimated glomerular filtration rate > 90) and C-reactive protein (< 3). She, however, had a low haemoglobin of 87.0 g dl^–1^, slightly low potassium of 3.3 mmol l^–1^, raised white cell count of 12.4 × 10^1^ l^–1^ with a neutrophil count of 11.53 × 10^1^ l^–1^ and a plasma lactate of 5 mmol l^–1^. Urine dipstick showed ketones +3 and blood +4 with no leukocytes or nitrites.

She was treated conservatively with i.v. fluids and potassium replacement, antiemetics and analgesia that she seemed to respond to. Following i.v. fluids, blood gas analysis showed a pH of 7.3, lactate of 2.12 and base excess of 2.5.

## Investigation/imaging

**Figure 1.**
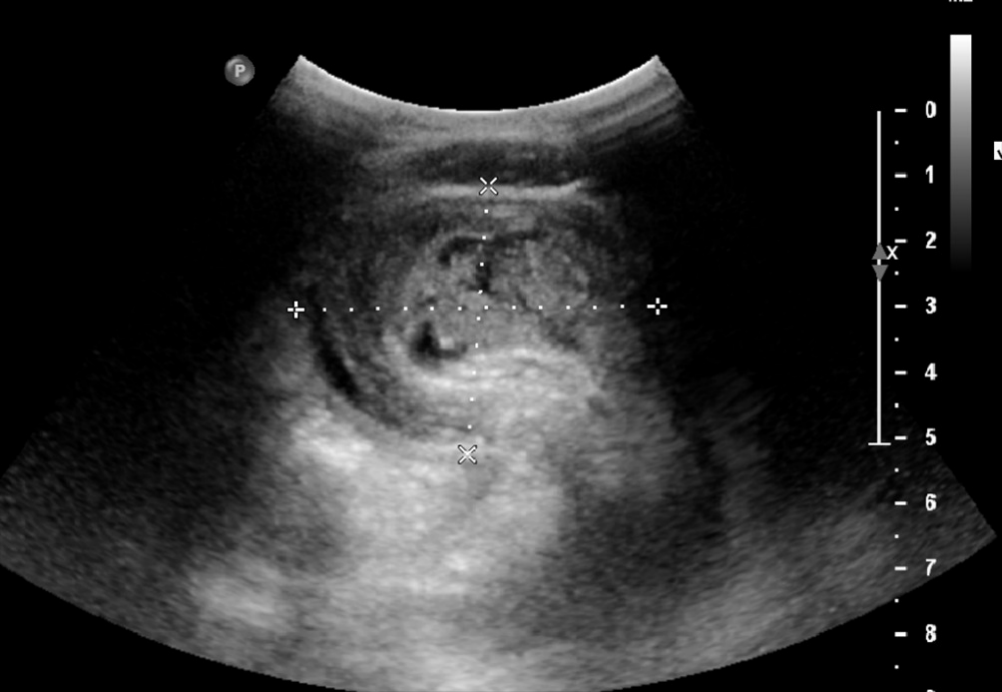
Cross-sectional ultrasound showing small bowel intussusceptionwith a “targetoid” appearance measuring 5.5 × 4.1 cm.

**Figure 2.**
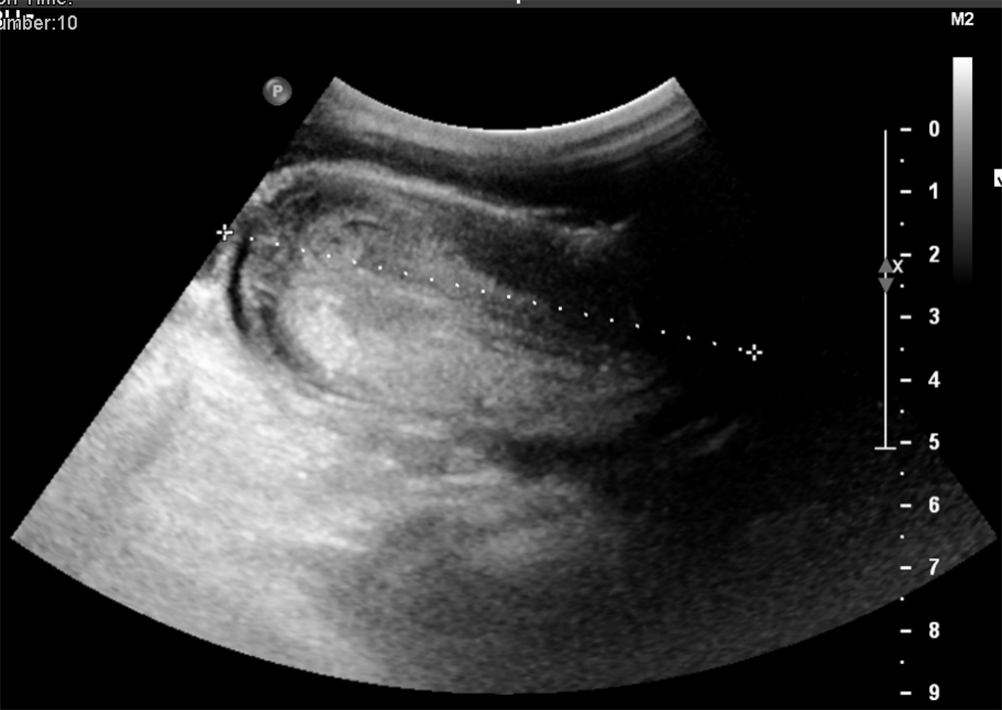
Longitudinal view of intussuscepted bowel on ultrasound measuring 8.2 cm.

Initial investigation was with an ultrasound scan, which showed two bowel-related masses within the lower abdomen, one lying in the midline inferior to the umbilicus and the second within the right iliac fossa (RIF). A “lumen in a lumen” appearance (Figures 1 and 2) was seen and some free fluid was present in the RIF. This was suspicious for intussusception and prompted further investigation with a CT scan.

CT scan of the abdomen showed multiple very oedematous loops of small bowel centrally within the lower abdomen immediately superior to the bladder, with no enhancement of these oedematous loops with i.v. contrast. There was also a small collection of free intraperitoneal gas anteriorly within the lower abdomen suggestive of bowel perforation. The rest of the abdominal viscera (stomach, pancreas, spleen, liver and gallbladder) and adnexal structures were normal. There was no lymphadenopathy.

## Treatment

The patient underwent an emergency laparotomy and had a right hemicolectomy and washout with side-to-side anastomoses of the small bowel and colon performed. Intraoperatively, there appeared to be a 40-cm ileoileal intussusception extending to the ileocaecal valve and causing distal small bowel obstruction in the RIF. There was also a 3 × 4 cm firm lump at the ileocaecal valve and a dilated segment of thickened, ischaemic distal ileum. There was no faecal contamination. The affected portion of the bowel was resected (40 cm) and a side-to-side anastomosis was performed.

Postoperatively, she was transferred to the surgical high dependency unit and stepped down to the ward a day later.

## Outcome/follow-up

The patient made a good recovery postoperatively. She tolerated oral intake, passed bowel motions and was subsequently discharged 7 days after admission. On histological analysis of the resected portion of bowel, there were no identifiable tumours, polyps or malignant lesions macroscopically. The appendix was also normal. On microscopic examination, ischaemic changes were present.

## Discussion

Intussusception, defined loosely as telescoping of one part of bowel into another adjacent part of the bowel, is a common differential in children presenting with acute onset abdominal pain. In adults, however, intussusception is quite rare, accounting for only 5% of the total cases of intussusception and 1–5% of cases of bowel obstruction.^1–3^ 90% of cases occur in the small or large bowel and only about 50% are diagnosed preoperatively.^3^

In most cases, presentation is with one or more of the following symptoms: colicky abdominal pain, abdominal distension, a palpable abdominal mass, constipation, bloody stools and absent bowel sounds.^1,3,4^ Only about 50% of the total cases of intussusception in adults is diagnosed preoperatively.^1,3^ Unlike in children where causes of intussusception are mostly primary and can be managed conservatively with pneumatic or hydrostatic reduction, adult cases tend to be due to pathological conditions, with a large percentage requiring surgical intervention.^3,4^

Based on where they occur, intussusception can be categorized into (i) ileocolic; (ii) colocolic; (iii) enteroenteric; and (iv) ileocaecal.^1,3,4^ Intussusception involving the small bowel can be due to benign lesions such as polyps in the gastrointestinal tract, Meckel’s diverticulum, inflammatory lesions and adhesions, with malignant lesions being responsible for up to 30% of cases. This is in contrast to cases occurring in the large bowel in which malignant lesions make up approximately 66% of cases.^3^ Between 8% and 20% of the total cases in adults are idiopathic with no identifiable cause.^5^

Diagnosis of intussusception is difficult in adults because it is rarely suspected and presentation is often variable.^1,3^ Imaging plays a vital role in the pre-operative diagnosis.

Abdominal plain films can serve as a good first-line investigation, as most cases present with signs of abdominal obstruction.^1^ Distended loops of bowel can be picked up on plain films.^3^ Intussusception is also widely diagnosed on ultrasound scans, showing the characteristic “bullseye” or “target” sign on transverse view (Figure 1) and pseudo-kidney sign on longitudinal views.^2,3,5^ Ultrasonography is particularly beneficial as it is of relatively low risk and non-invasive with a false-negative rate of close to zero.^6,7^

**Figure 3.**
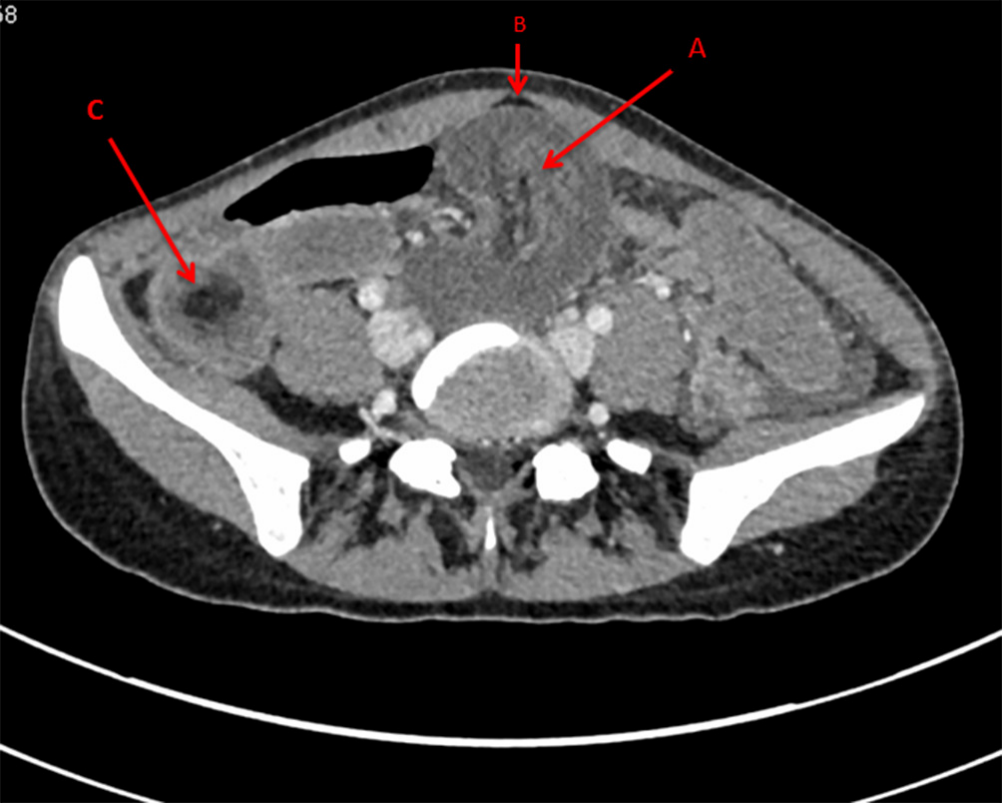
Reniform mass representing non-enhancing oedematous loops of the small bowel. (b) Small collection of free intraperitoneal gas. (c) Intussusception of the terminal ileum into the caecum appearing as a “Target sign”.

Abdominal CT is currently the most sensitive scan for diagnosing intussusception in adults, with a diagnostic accuracy of 58–100%.^1,3^ Features on CT scan include a “target” sign representing triangular fat density of the mesentery of the intussusceptum surrounding a soft tissue mass, or a “sausage”-shaped mass, which occurs in more advanced stages and represents alternating layers of low and high attenuation of the mesenteric fat and bowel wall, respectively.^3,8^ If ischaemia has occurred, then a reniform (kidney-shaped) mass can be seen as a result of oedema and necrosis (Figure 3).^8^ CT scan provides an added benefit during staging in cases where the suspected cause is a malignancy.^1^

Other investigations that can be used are barium enemas (mostly used in children) and upper gastrointestinal contrast studies, which may show “coil spring” appearance (representing barium in the lumen of the intussusceptum and the intraluminal space) or “stacked coin” defects, which signify bowel obstruction in the small or large bowel.^3,4^

In adults, 70–90% of patients will undergo surgical resection as a definitive treatment.^1^ This is because most cases have pathological causes, requiring resection and histological analysis.^3^ Also, owing to the delayed diagnosis, bowel ischaemia most likely occurs and resection of the infarcted bowel is often necessary.^3^ Different views still exist regarding whether or not to attempt reduction of the intussuscepted portion of the bowel before resection.^2,4^ In general, individuals with colocolic intussusception tend to have resection without reduction owing to the risk of malignancy in order to minimize the theoretical risk of intraluminal seeding.^2,5^ In those with small bowel intussusception, reduction can be attempted before resection if there are no signs of bowel ischaemia or suspected malignancy.^5^

## Learning points

1. Intussusception in adults poses a diagnostic challenge, and early imaging is vital in the prompt diagnosis of suspected cases.
2. Abdominal CT is the most sensitive imaging modality for diagnosing such cases but other modalities such as X-rays and ultrasonography can also be utilized.
3. Characteristic signs such as “target” and “pseudokidney” signs on ultrasound and reniform masses on CT scan can aid in diagnosis.
4. Treatment in most cases is with surgery and usually involves resection of the affected portion of the bowel.

## Consent

I confirm that written informed consent for the case (including accompanying images, case history and data) to be published was obtained from the patient for publication of this case report.
